# Is Hydroxychloroquine Useful for the Treatment of Cutaneous Manifestations of Idiopathic Inflammatory Myopathies? A Systematic Review

**DOI:** 10.3390/ph18091293

**Published:** 2025-08-29

**Authors:** Jucier Gonçalves Júnior, Jamily Izabel Alves dos Santos, Samuel Katsuyuki Shinjo

**Affiliations:** 1 School of Medicine, Universidade Federal do Cariri (UFCA), Barbalha 63180-000, Ceará, Brazil; jamily.izabel@aluno.ufca.edu.br; 2 School of Medicine, Universidade Regional do Cariri (URCA), Crato 63105-000, Ceará, Brazil; 3 Division of Rheumatology, Faculdade de Medicina FMUSP, Universidade de São Paulo, São Paulo 01246-903, São Paulo, Brazil; samuel.shinjo@usp.br

**Keywords:** chloroquine, dermatomyositis, hydroxychloroquine, inflammatory myopathy, myositis

## Abstract

**Background/Objectives**: Hydroxychloroquine (HCQ) is frequently used to manage cutaneous manifestations associated with idiopathic inflammatory myopathies (IIMs). Nevertheless, the literature lacks consensus regarding the efficacy and safety of drugs for these manifestations. **Methods**: A systematic literature review was conducted in accordance with the Preferred Reporting Items for Systematic Reviews and Meta-Analyses (PRISMA) protocol. The search spanned the period from January 1958 to April 2025 across the following databases: PubMed, Scopus, Web of Science, Cochrane Library, PsycInfo, and Virtual Health Library. Articles were included if they contained at least one of the specified descriptors in the title or abstract; were published in English, Portuguese, or Spanish; and addressed the use of HCQ or chloroquine (CQ) in the context of skin manifestations of IIMs. Review articles, experimental studies, and short communication articles were excluded. **Results**: Among the 319 patients assessed, the majority were women diagnosed with dermatomyositis or juvenile dermatomyositis. The most prevalent cutaneous manifestations were Gottron’s papules and diffuse erythematous lesions. The most frequent extracutaneous manifestations were muscle weakness and arthritis/arthralgia. HCQ was administered in 74% of the cases, with dosages ranging from 200 to 400 mg/day and a follow-up duration of 26 months. In most cases, it is administered in conjunction with glucocorticoids. Adverse effects were observed in less than 9% of the patients, with myalgia, skin lesions, and photosensitivity being the most common. However, the use of CQ has not been documented in any of these studies. **Conclusions**: Although there are studies in the literature using HCQ as part of the treatment of cutaneous manifestations in patients with IIMs, longitudinal studies with larger sample sizes and more robust methods are required to evaluate the applicability of HCQ in the treatment of IIMs.

## 1. Introduction

Idiopathic inflammatory myopathies (IIMs), or systemic autoimmune myopathies, are a heterogeneous group of diseases affecting the cardiovascular and respiratory systems, gastrointestinal tract, muscles, and skin [1,2]. IIMs include dermatomyositis (DM), clinically amyopathic DM, anti-synthetase syndrome (ASyS), polymyositis (PM), immune-mediated necrotizing myopathy (IMNM), juvenile DM, and inclusion body myositis (IBM) [3,4,5].

Cutaneous manifestations are common and can occur in up to 80% of DM cases, leading to deterioration in quality of life, and may follow muscular disease for months or years [6,7,8,9]. Cutaneous manifestations in IIMs may be acute if they occur up to six months after disease onset and indicate disease activity, or they may be chronic or scarring if they persist for six months after initiation of therapy. Acute lesions include Gottron’s papules, heliotrope rash, shawl sign, “V-neck” sign, Holster’s sign, calcinosis cutis, and cuticle hypertrophy [10,11,12].

The most commonly used treatment for the cutaneous symptoms of IIMs is based on the use of immunosuppressive drugs, with prednisone being the therapy of choice for several decades, often in conjunction with corticosteroid-sparing drugs such as methotrexate, chloroquine (CQ), and hydroxychloroquine (HCQ) with complete response, especially to the use of methotrexate in conjunction with prednisone or other immunosuppressive drugs such as azathioprine or mycophenolate mofetil [13,14,15,16,17,18,19,20,21]. Currently, new therapies have been proposed for the treatment of IIMs, particularly in cases refractory to corticosteroids. Notable among these are rituximab, intravenous immunoglobulin (IVIG), and Janus kinase-STAT (JAK) inhibitors (iJAK). IVIG has demonstrated efficacy in difficult-to-treat cases, whereas iJAK inhibitors have shown good results in improving pulmonary, muscular, and cutaneous symptoms. However, to date, evidence is limited and contradictory, particularly in relation to the treatment of skin manifestations of DM and other IIMs, owing to the rarity of these diseases and the lack of controlled clinical trials [1,17].

CQ and HCQ are antimalarials that were introduced into clinical practice more than six decades ago. With the advancement of medical and pharmacological knowledge, their use has extended to the treatment of various rheumatic and dermatologic diseases, including systemic lupus erythematosus and rheumatoid arthritis, owing to their immunomodulatory and anti-inflammatory properties [13]. In the context of IIMs, the literature describes the use of HCQ and CQ in outpatient settings, particularly for the treatment of skin manifestations associated with DM, to improve symptoms such as photosensitivity, rashes, and erythematous lesions [5,9,14,15,16,17,18,19,20,21,22,23,24,25,26,27,28,29]. However, there is no consensus in the literature regarding the efficacy and safety of this drug in the skin manifestations of IIMs.

Therefore, this study aimed to conduct a qualitative systematic review of the literature based on the following guiding question: What evidence does the current scientific literature provide for clinical practice regarding the use of HCQ/CQ in the treatment of cutaneous manifestations of IIMs?

## 2. Methods

### 2.1. Literature Review

A qualitative systematic review of the literature was conducted according to the PRISMA protocol using six electronic databases: Virtual Health Library, PubMed, Cochrane library, Web of Science, Scopus, and PsycInfo. The electronic searches utilized the Medical Subject Headings (MeSH) descriptors: #1 “Hydroxychloroquine”; #2 “Chloroquine”; #3 “Idiopathic inflammatory myopathies”; #4 “Polymyositis”; #5 “Dermatomyositis”; #6 “Anti-synthetase syndrome”; #7 “Immune-mediated necrotizing myopathy”; #8 “Myositis, inclusion body”. These descriptors were combined using the Boolean Operator “AND”.

The search period was extended from January 1958 to April 2025, with 1958 defined as the starting year, as it corresponded to the publication date of the oldest article found in the databases. This review applied the PICO framework, in which “P” represents patients with IIMs; “I”: presence of treatment with HCQ or CQ in IIM; “C” refers to groups without treatment with HCQ or CQ; and “O” denotes the impact of treatment on the skin.

### 2.2. Data Collection

The search strategy included the following combinations: #1 AND #3; #1 AND #4; #1 AND #5; #1 AND #6; #1 AND #7; #2 AND #3; #2 AND #4; #2 AND #5; #2 AND #6; #2 AND #7 (Supplementary Materials).

Data collection was conducted in May and June 2025. Manuscripts were selected primarily through an analysis of their titles and abstracts. Two researchers independently collected the data to ensure the reliability of the results, and disagreements were addressed by a third senior researcher. The senior researcher followed the following protocol: (i) evaluated the question; (ii) evaluated the original paper/file or information; and (iii) compared the information with the current guidelines for IIMs to assess the plausibility of the information. Each article in the sample was read in full, and the extracted data were entered into a spreadsheet comprising Table 1 and Table 2.

Table 1 and Table 2 summarizes the following information: author (year), country, study type, study quality, gender, ethnicity, age (mean), IIM subtype, myalgia, muscle weakness, cutaneous manifestations such Gottron’s papules, heliotrope rash, shawl sign, “V-neck” sign, cuticle hypertrophy, calcinosis, diffuse rash, holster sign, and vasculopathy; arthralgia/arthritis, Raynaud phenomenon, interstitial lung disease, gastrointestinal involvement, HCQ treatment regimen, associated medications, assessment time (months) and outcome.

Tools from the National Heart, Lung, and Blood Institute, including the Study Quality Assessment Tool, were used to analyze the quality of each study (Table 1). The Quality Assessment Tool for Observational Cohort and Cross-Sectional Studies and the Quality Assessment Tool for Case Series Studies were used for observational cross-sectional and cohort studies, and case reports and series, respectively. These tools classify studies as “Good,” “Fair” or “Poor” based on the presence or absence of methodological elements relevant to each type of study.

Articles that met all items on the National Heart, Lung, and Blood Institute questionnaire were categorized as “good”. The absence of any criterion that did not affect the quality of the study (e.g., adherence to a well-established protocol, sample size, sampling error, and errors in data interpretation) was categorized as “fair”. The presence of one or more of the above criteria categorized articles as “poor”.

### 2.3. Eligibility Criteria

Articles were analyzed based on the following eligibility criteria: inclusion of at least one combination of the terms described in the search strategy in the title or abstract; complete online access to the full text, written in English, Portuguese, or Spanish; and addressing the use of HCQ or CQ in the treatment of cutaneous manifestations of IIMs.

Studies with unclear methodologies, patients with cutaneous manifestations of other rheumatic diseases that were not myopathies or overlap syndromes, theses, dissertations, editorials, letters, or reviews were excluded. Manuscripts listed in more than one database were counted only once, and duplicates were removed using the Mendeley reference manager (https://www.mendeley.com/search/, accessed on 31 May and 30 June 2025).

### 2.4. Ethical Issue

As this was a systematic review of the literature, Resolution 510/16 of the National Health Council (CNS; acronym in Portuguese) did not require approval from the Human Research Ethics Committee. This review was registered on the Prospero platform under the number CRD420251069238.

## 3. Results

**Figure 1:**
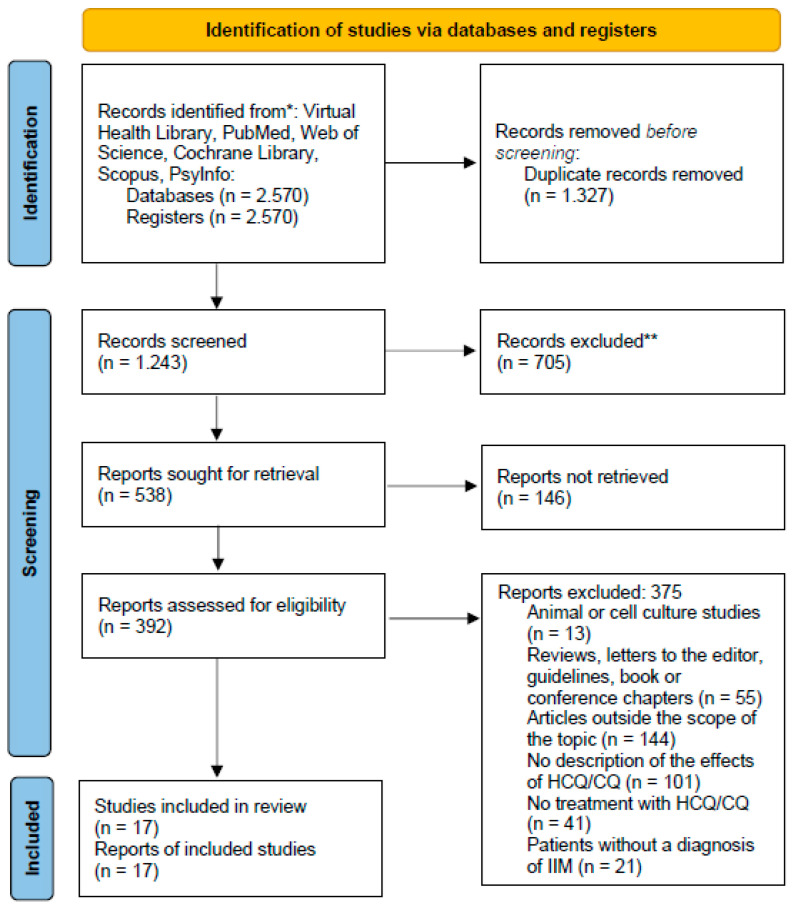
PRISMA flow diagram [Adapted].

Of the 2570 articles found in the databases, 17 met the eligibility criteria (Figure 1).

The sample consisted mainly of case series and reports (82.4%), followed by cohorts (17.6%). Regarding geographical location, studies were predominantly conducted in North America (41.2%), followed by Asia (29.4%), Europe (23.5%), and South America (5.9%). Most studies were classified as “good” (76.5%) and the others as “fair” (23.5%).

Of the 319 people examined, most were female (68.7%) and Caucasian (85.0%). The median age of the patients was 38 years (range, 5–80 years). Juvenile DM was the most common IIMs, with 185 (58.0%) patients, followed by DM (124/320, 38.9%), and juvenile PM (9/320, 2.8%). Only one had ASyS.

Muscle weakness (89.0%), Gottron’s papules (56.4%), and extensive skin rashes (54.9%) were the most common manifestations. More than one-third of the patients had extracutaneous manifestations, such as myalgia (69.0%), arthralgia (35.1%), and arthritis (28.2%). Interstitial lung disease occurred in only one patient with ASyS.

HCQ was used in 74.0% of patients with skin manifestations, especially in patients with DM. Improvement in the condition was observed in 72.9% of the cases. Most cases were patients with ASyS, juvenile DM, juvenile PM, and severe DM with extensive skin, lung, and joint involvement. The doses used ranged from 200 to 400 mg/day.

The most common concomitant medication with HCQ was prednisone (64.8%). Other concomitant medications included methotrexate (11.7%), mycophenolate mofetil (6.8%), azathioprine (4.2%), intravenous immunoglobulin (3.4%), prednisolone (0.4%), cyclosporine (0.8%), tacrolimus (0.8%), and rituximab (0.4%). The mean follow-up time in the studies was 26.7 months.

HCQ was used as monotherapy in only one case of mild DM without significant pulmonary, muscular, or cardiac involvement [24].

Adverse reactions occurred in 29 patients (9.1%). The following were reported: (i) exacerbation of weakness (10.3%), including myopathy; (ii) exacerbation of skin symptoms (89.7%), including photosensitivity; (iii) peripheral edema (6.9%); (iv) gastrointestinal symptoms (10.3%); and (v) retinal toxicity (3.4%) and anorexia (3.4%).

The use of CQ has not yet been reported; therefore, the data in this review refer exclusively to HCQ. It is also noteworthy that there are no reports of studies in which HCQ was administered to patients with PM, IBM, or IMNM.

For heuristic reasons, we decided to divide the results into two topics: “efficacy of HCQ in the treatment of cutaneous manifestations of IIMs” and “safety and adverse effects of HCQ in the treatment of cutaneous manifestations of IIMs”.

## 4. Discussion

### 4.1. Efficacy of HCQ in the Treatment of Cutaneous Manifestations of IIMs

HCQ is an antimalarial drug with anti-inflammatory properties [13]. Its mechanism of action involves influencing the acidification and fusion of lysosomes, endosomal trafficking, and inhibition of autophagy. As a result, antigen presentation and apoptosis of memory T cells occur. In addition to these effects, HCQ has been shown to inhibit the signaling of toll-like receptors 7 and 9 as well as the cGAS–STING pathway, which are responsible for the production of IFN-α and IFN-β [19].

These effects may help to understand how and why HCQ has been used in the treatment of the cutaneous manifestations of DM, as the pathophysiology is associated with the presence of IFN, which plays an important role in the inflammatory process affecting the skin and muscles [1,6].

Photosensitivity, Gottron’s papules, and heliotrope were the most common manifestations of IIMs in our analysis. HCQ as monotherapy or in combination with other immunosuppressants (e.g., glucocorticoids, methotrexate, mycophenolate mofetil, or rituximab) was the drug of choice for the treatment of skin manifestations in 74% of cases [8,14,15,18,19,20,21,22], with improvement of symptoms in 72% of the sample [8,9,14,15,16,18,19,20,21,22,23,24,25,26]. However, this improvement could not be attributed to HCQ as an adjuvant or to the use of other immunosuppressants in combination.

A cohort study conducted by Vignesh et al. [25] described four cases of patients with juvenile DM who achieved remission of skin disease by combining HCQ, mycophenolate mofetil, and methotrexate, underlining the role of HCQ in combined therapies. In another cohort study conducted by Sato et al. [15], it was observed that skin symptoms resolved satisfactorily in patients treated with HCQ, although two patients died due to complications of IIMs. Reports have demonstrated the efficacy of HCQ monotherapy in some cases. Roesler and Jenkins [24] reported the case of a 68-year-old patient with DM who was treated with HCQ monotherapy. The patient had Gottron’s papules and poikilodermatous changes on the face, neck, and upper body, as well as subtle skin indurations on the face and cape area, without organic involvement or marked muscle weakness.

Despite apparently favorable results, the efficacy of HCQ as a monotherapy is limited, and its isolated use does not appear to be sufficient to control skin manifestations and overall disease activity [27]. Compared with conventional therapies used to treat the cutaneous manifestations of IIMs, HCQ has been observed to provide satisfactory results as monotherapy in cases of lower severity [24]. However, patients with refractory conditions had to be treated with stronger immunosuppressive drugs such as methotrexate, mycophenolate mofetil, and methylprednisolone [9,16,28]. In addition, adverse skin reactions associated with the use of HCQ were noted, which in some cases led to the treatment being discontinued and replaced with other therapies, such as prednisone, methotrexate, and topical corticosteroids [14,23].

Furthermore, most studies were case studies or case series [8,9,14,15,16,18,19,20,21,22,23,24,25,26,28,29], which only guarantees a retrospective analysis. In addition, it is important to emphasize that the efficacy of drugs cannot be adequately assessed owing to differences in treatment regimens and the lack of studies with adequate follow-up designs and sample sizes.

### 4.2. Safety and Adverse Effects of HCQin the Treatment of Cutaneous Manifestations of IIMs

Although HCQ can be used to treat skin manifestations of IIMs, its use may be associated with adverse effects. Dermatologic reactions, including maculopapular, erythematous, or urticarial drug eruptions and cutaneous hyperpigmentation, are common [14]. In addition, manifestations in other organs may also occur, such as retinopathy, gastrointestinal symptoms, and muscle weakness [13,19].

Furthermore, it is possible that the different effects of HCQ are related to the differential actions of specific nucleic acid recognition pathways depending on the autoantibody subsets involved [19].

In this review, adverse effects were observed in 9% of patients, including worsening of muscle weakness, worsening of skin symptoms, edema, and gastrointestinal symptoms such as dyspepsia, macular toxicity, and anorexia, especially in DM [13,14,16,20,23,27,29].

Cutaneous reactions have been frequently reported, including morbilliform eruptions, hyperpigmentation, pruritus, Steven-Johnson syndrome, toxic epidermal necrolysis, and acute generalized exanthematous pustulosis [23]. In a study by Wolstencroft et al. [19], 23 (20.7%) of 111 patients treated with HCQ experienced worsening skin symptoms during DM treatment. Similarly, Ripka et al. [14] reported a series of three patients with DM who developed adverse skin reactions after starting HCQ treatment, with symptoms improving after discontinuation of the drug, suggesting a possible causal relationship.

It also remains unclear whether some skin symptoms, such as pruritus, are due to disease activity or pharmacoderma.

Another relevant adverse effect in rheumatology practice is macular toxicity, which is generally caused by HCQ doses higher than 5 mg/kg/day, use of HCQ for more than 10 years, use of HCQ in combination with tamoxifen, or the use of HCQ in patients with chronic kidney disease. Brandao and Wolfe [13] described the case of a 15-year-old patient with juvenile DM who developed maculatoxicity after three years of HCQ use without exceeding the recommended therapeutic dose, emphasizing that this adverse effect can also occur with short- to medium-term treatments [13].

Finally, the lack of information on the dose used, follow-up [9,22], details such as serum levels of creatine phosphokinase and aldolase (in suspected HCQ-induced myopathy), and the autoantibody profile of IIMs treated in the trials prevents a better understanding of the safety of the drug [22].

The limitations of this review include (i) the retrospective, cross-sectional nature and small sample size of the selected studies; (ii) the inability to perform a meta-analysis; (iii) the lack of homogenization of assessments using standardized scores for skin assessment, such as the Cutaneous Dermatomyositis Disease Area and Severity Index (CDASI) or Cutaneous Assessment Tool (CAT); and (iv) the lack of homogenization of therapeutic approaches (no consensus on the use of dose and combinations of medications with HCQ) for adults and children with IIMs.

## 5. Conclusions

Although there are reports in the literature on the use of HCQ for the treatment of cutaneous manifestations of IIMs, the available evidence is insufficient to fully support this indication. HCQ did not appear to have a significant effect on skin activity in patients with IIMs, particularly in those with DM and juvenile DM. The literature indicates that its use is associated with more intensive immunosuppressive therapies, suggesting a possible trend towards its use as an adjunct to enhance established or investigational therapies. Side effects occur in a minority of patients, with myopathy and worsening skin symptoms being the most common, whereas macular toxicity is rare.

Longitudinal studies with large sample sizes and robust methodologies are needed to assess the true applicability of HCQ in the treatment of IIMs, particularly DM and juvenile DM.

## Figures and Tables

**Table 1:** Main characteristics of the studies included in the review regarding the clinical-epidemiological aspects of IIM with HCQ.

Author [Ref] - Country (year) - Study - Quality	Female/Total Ethnicity	Age (Years)	IIM Subtype	Gottron’s Papules, Heliotrope Rash, shawl Sign, “V-Neck” Sign, Cuticle Hypertrophy, Calcinosis, Diffuse Rash, Holster Sign, Vasculopathy	Myalgia, Muscle Weakness, Arthralgia, Arthritis	RP, ILD, GI Involvement
Caporali et al. [21] - Italy (2003) - Case report - Good	1/1 Caucasian	46	DM	N, N, N, N, N, N, 1, N, N	1, 1, N, N	N, N, N
Sato et al. [15] - Brazil (2009) - Cohort - Fair	117/187 Caucasian	7	178 JDM 9 JPM	157, 157, NA, NA, NA, 188, NA, NA, NA	118, 181, 103, 85	85, 178, 179 (dysphagia)
Ishaque et al. [22] - Pakistan (2011) - Case report - Fair	1/1 Parda	14	JDM	1, N, N, N, N, N, 1, 1, N	1, 1, 1, 1	1, Y, N
Brandao & Wolfe [13] - Switzerland (2016) - Case report - Fair	1/1 Caucasian	15	JDM	NA, NA, NA, NA, NA, NA, NA, NA, NA	NA, NA, NA, NA	NA, NA, NA
Antonopoulos & Liossis [8] - Greece (2018) - Case report - Good	1/1 Caucasian	68	DM	1, 1, 1, N, N, N, N, 1, 1	1, N, 1, N	N, NA, NA
Wolstencroft et al. [19] - USA (2018) - Cohort - Fair	89/111 71 Caucasian, 13 Asian, 3 Oceanian, 2 Afro-American, 22 Hispanic/Latino	49.9	111 DM	NA, NA, NA, NA, NA, NA, NA, NA, NA	93, 93, NA, NA	NA, NA, NA
Dziwis et al. [16] - USA (2019) - Case report - Good	1/1 Caucasian	38	DM	NA, NA, NA, NA, NA, NA, 1, N, N	1, 1, 1, 1	NA, NA, NA
Chiu et al. [26] - Philippines (2020) - Case report - Good	0/1 Caucasian	36	DM	1, N, N, 1, N, 1, N, N, 1	1, 1, NA, NA	NA, N, Dysphagia
Franciosi et al. [28] - USA (2020) - Case report - Good	1/1 Hispanic	80	DM	N, N, 1, N, N, N, N, N, N	N, N, N, N	N, N, N
Roesler & Jenkins [24] - Canada (2021) - Case report - Good	1/1 Caucasian	68	DM	1, N, N, 1, 1, N, N, N, N	N, N, N, N	N, N, N
Rosa et al. [29] - Sri Lanka (2021) - Case report - Good	1/1 Asian	45	ASyS	N, N, N, N, N, N, 1, N, N	1, 1, N. N	N, 1, N
Nguyen et al. [18] - USA (2021) - Case report - Good	0/1 Parda	57	DM	1, 1, N, N, N, N, N, N, 1	NA, NA, 1, 1	1, 1, N
Ripka et al. [14] - USA (2023) - Case series - Good	2/3 Caucasian	47,6	3 DM	2, N, N, 2, N, 1, 1, 2, N	NA, NA, 1, 1	N, N, 1 (GERD)
Vignesh et al. [25] - India (2023) - Cohort - Good	1/4 Indian	6.75	JDM	4, 4, 1, NA, NA, NA, 4, N, 1	1, 3, 1, 1	N, 2, N
Pruneda et al. [23] - USA (2024) - Case report - Good	1/1 Caucasian	75	DM	1, 1, 1, 1, NA, NA, NA, NA, NA, NA	NA, NA, NA, NA	NA, 1, Dysphagia
Wulandari et al. [20] - Indonesia (2024) - Case report - Good	1/1 Caucasian	7	JDM	1, 1, N, N, N, 1, N, N, N	1, 1, N, N	N, 1, N
Dens et al. [9] - Belgium (2025) - Case report - Good	0/2 Caucasian	59	2 DM	1, N, N, 1, N, N, 1, N, N	1, 1, N, N	N, N, N

Legend: ASyS: Anti-synthetase syndrome; DM: dermatomyositis; JDM: juvenile dermatomyositis; ILD: interstitial lung disease; GERD: gastroesophageal reflux disease; GI: gastrointestinal; IIM: idiopathic inflammatory myopathies; N: absent; NA: not available; RP: Raynaud’s phenomenon.

**Table 2:** Main characteristics of the studies included in the review regarding the treatment aspects of IIM with HCQ.

Author [Ref]	HCQ Treatment Regimen (mg/day)	Associated Medications	Assessment Time (mo)	Outcome
Caporali et al. [21]	400	PRED	26	Resolution of the skin manifestations
Sato et al. [15]	200	PRED or MTX	42	Resolution of the skin manifestations. Two patients using HCQ died due to complications of IIM
Ishaque et al. [22]	NA	PRED	NA	Resolution of the skin manifestations
Brandao & Wolfe [13]	200 mg/d	CsA	36	Resolution of the skin manifestations, but developed macular toxicity with HCQ
Antonopoulos & Liossis [8]	200 for three days follow up to 400	PRED + FK cream, MP + IGIV RTX	06	Resolution of the skin manifestations
Wolstencroft et al. [19]	200–400 (93) or 400–800 (4)	58 PRED, 21 MTX,11 MMF, 8 AZA, 6 IGIV	40	Twenty-three patients developed HCQ-related rash, including exacerbation of DM (5), photosensitive rash (2), and nonspecific, erythematous, diffuse, and pruritic rash (most), as well as symptoms such as increased muscle weakness (3), peripheral edema (2), and gastrointestinal symptoms (2). The remaining patients did not develop a rash. Fifty two patients showed remission of the skin disease.
Dziwis et al. [16]	400	Triamcinolone ointment/cream	11	There was no improvement in the condition, with discontinuation of HCQ
Chiu et al. [26]	200	MP, hydrocortisone	2	Partial improvement in skin manifestations, associated with weight gain and improved muscle strength
Franciosi et al. [28]	200	MTX	95	Resolution of the skin manifestations
Roesler & Jenkins [24]	200	N	48	Resolution of the skin manifestations
Rosa et al. [29]	200	PDN, AZA, CFF	36	Resolution of the skin manifestations
Nguyen et al. [18]	300	PRED, FK	05	Improvement of the skin condition with partial improvement of the ILD condition
Ripka et al. [14]	400	Diltiazem, PRED, 1 MTX, 1 AZA, 1 MMF, 1 CsA	10	The three cases were caused by HCQ use, with improvement after stopping use.
Vignesh et al. [25]	1–3 mg/kg/day	MP, MTX	39	Resolution of the skin manifestations
Pruneda et al. [23]	400	N	06	The DM skin condition was unresponsive to HCQ, with discontinuation. It was treated with RTX and MMF
Wulandari et al. [20]	100	PRED, MTX	09	Resolution of the skin manifestations
Dens et al. [9]	400	MMF, MP IGIV	NA	Resolution of the skin manifestations

Legend: AZA: azathioprine; CFF: cyclophosphamide; CQ: chloroquine; CsA: cyclosporine; DM: dermatomyositis; FK: tacrolimus (also known as FK506); HCQ: hydroxychloroquine; IVIG: intravenous immunoglobulin; MDA5: melanoma differentiation-associated protein 5; MMF: mycophenolate mofetil; IIM: idiopathic inflammatory myopathies; MTX: methotrexate; mo: months; N: absent; NA: not available; PDN: prednisolone; PRED: prednisone; RTX: rituximab.

## Data Availability

All data analyzed in this study are included in this published article and Supplementary Materials.

## Supplementary Materials

The following supporting information can be downloaded at: https://www.mdpi.com/article/10.3390/ph18091293/s1.
