# Supplementary material for: Is Hydroxychloroquine Useful for the Treatment of Cutaneous Manifestations of Idiopathic Inflammatory Myopathies? A Systematic Review
Source: Pharmaceuticals (Basel). 2025 Aug 29;18(9):1293. doi: 10.3390/ph18091293 (PMC12472818; doi:10.3390/ph18091293)
Supplement: Supplementary file 1 [file pharmaceuticals-18-01293-s001.zip › pharmaceuticals-3731640-supplementary.pdf]

RESEARCH PROTOCOL

- Month of collection: May 31 to June 2, 2025

- Period of publication of the Periodicals: January 1, 1958 and April 17, 2025

- MeSH terms:

#1 “Hydroxychloroquine” #2 “Chloroquine” #3 “Idiopathic inflammatory myopathies”

#4 “Polymyositis” #5 “Dermatomyositis” #6 “Antisynthetase syndrome”

#7 “Immune-mediated necrotizing myopathy” #8 “Myositis, Inclusion Body”

Databases:

1) Virtual Health Library (VHL)

(N° of articles found: 291 | N° of articles selected for analysis: 30 | N° of articles excluded: 261)

2) PubMed

(N° of articles found: 365 | N° of articles selected for analysis: 12 | N° of articles excluded: 353)

3) Web of Science

(N° of articles found: 220 | N° of articles selected for analysis: 43 | N° of articles excluded: 177)

4) Cochrane Library

(N° of articles found: 11 | N° of articles selected for analysis: 4 | N° of articles excluded: 7)

5) Scopus

(N° of articles found: 1.683 | N° of articles selected for analysis: 303 | N° of articles excluded: 1.380)

6) PsycInfo

(N° of articles found: 0 | N° of articles selected for analysis: 0 | N° of articles excluded: 0)

#1 AND #3	#1 AND #4	#1 AND #5	#1 AND #6	#1 AND #7	#1 AND #8
#2 AND #3	#2 AND #4	#2 AND #5	#2 AND #6	#2 AND #7	#2 AND #8

Source: Authors (2025)
